# Hepatic vein-derived factors may affect pulmonary arteriovenous malformations after single ventricle palliation by modulating vascular cell behavior

**DOI:** 10.1038/s41598-025-25523-1

**Published:** 2025-11-24

**Authors:** Laura Yuriko González-Teshima, Keisuke Hakamada, Kozue Murata, Reiko Nakagawa, Shiro Baba, Yujiro Ide, Maiko Okamura, Akio Ikai, Tadashi Ikeda, Kenji Minatoya, Masaya Hagiwara, Masaya Ikegawa, Hidetoshi Masumoto

**Affiliations:** 1 Department of Cardiovascular Surgery, Graduate School of Medicine, Kyoto University, 54 Kawara-cho, Shogoin, Sakyo-ku, Kyoto, 606-8507 Japan; 2 Clinical Translational Research Program, RIKEN Center for Biosystems Dynamics Research, Kobe, Japan; 3 Laboratory for Cell-Free Protein Synthesis, RIKEN Center for Biosystems Dynamics Research, Kobe, Japan; 4 Department of Pediatrics, Graduate School of Medicine, Kyoto University, Kyoto, Japan; 5 Department of Life and Medical Systems, Doshisha University, Kyotanabe, Japan; 6 Division of Pulmonary Hemodynamics Research, Department of Clinical Research, Research Support Center, Shizuoka General Hospital, Shizuoka, Japan; 7 Human Biomimetic System RIKEN Hakubi Research Team, RIKEN Center for Biosystems Dynamics Research, Kobe, Japan

**Keywords:** Arteriovenous malformations, Congenital heart defects, Prospective studies, Tissue engineering, Proteomics, Congenital heart defects, Proteomic analysis, Angiogenesis, Experimental models of disease, Paediatric research

## Abstract

Pulmonary arteriovenous malformations (PAVM) are a major surgical complication of univentricular heart therapy, significantly limiting patient survival. Interruption of hepatic venous drainage into the lungs has been long hypothesized to play a critical role in PAVM development by angiogenesis modulation. To understand human plasma role on PAVM development, we conducted a prospective study analyzing paired plasma samples from superior vena cava (SVC) and hepatic vein (HV) origin from 10 infants with congenital heart disease. Two and three dimensional in vitro arteriovenous models were implemented to compare the angiogenic potential of SVC and HV plasma, alongside untargeted shotgun proteomic profiling. Compared to HV, SVC plasma exhibited a pro-angiogenic profile in vitro. SVC proteome was enriched with pro-angiogenic S100 calcium-binding family proteins (S100A7/A8/A9/P). In contrast, HV plasma had unique expression of actin binding proteins (profilin 1/filamin A/actinin alfa 1), and upregulation of pathways associated with vascular stability and cell adhesion maintenance, suggesting a plausible counteracting role to SVC. Our results emphasize the importance of considering not only angiogenesis, but also extracellular mechano-transduction and vascular tone regulation in PAVM pathogenesis; and highlights the value of in vitro arteriovenous modeling to bridge the gap towards PAVM understanding and advancement of future therapeutical alternatives.

**Supplementary Information:**

The online version contains supplementary material available at 10.1038/s41598-025-25523-1.

## Introduction

Univentricular heart diseases, a broad group of cardiac structural abnormalities characterized by two atria connected to a functional univentricular chamber^1^ represent 7.7% of congenital heart diseases, the most common type of neonatal malformations^2^. Although palliative surgery with a total cavopulmonary connection (Fontan procedure) has significantly improved survival, the development of pulmonary arteriovenous malformations (PAVM) remains a major limitation to the completion and long-term success of univentricular heart disease therapy^3^.

PAVM are abnormal vessels that bypass the alveolo-capillary oxygenation bed, leading to “right-to-left” shunting, progressive hypoxia and ultimately death^4^. Over 70% of patients develop PAVM after superior cavopulmonary connection (SCPC) or bidirectional Glenn shunt, the transitional surgical step before Fontan completion^5^. SCPC interrupts hepatopulmonary circulation by exclusively directing blood from the superior vena cava (SVC) into the pulmonary vasculature, excluding hepatic vein (HV) flow and leading to PAVM development^1^.

PAVM are not limited to SCPC patients; they also occur in liver disease, such as hepatopulmonary syndrome (HPS), where abnormal intrapulmonary vascular dilatations develop despite normal cardiopulmonary anatomy^6^. Both anatomical (SCPC) and functional (liver disease) interruptions of hepatic venous flow to the lungs have been linked to PAVM formation; however molecular parallels between PAVM secondary to univentricular heart disease and liver dysfunction remain unclear. Clinical and animal experimental evidence supports a hepato-pulmonary interaction, possibly mediated by a short-half life, highly concentrated vascular modulator. This “hepatic factor”, present in HV blood, has been long believed to be the key to the pulmonary vascular remodeling in PAVM. Nevertheless, despite ongoing efforts, it remains unidentified, and PAVM pathogenesis and treatment remain unresolved^5^.

In vitro disease modeling and in particular, three-dimensional organ-on-a-chip (3D OoC) technologies, offer an invaluable alternative to study vascular pathology in highly physiologically relevant and controlled platforms^7^. Based on the hypothesis that HV derived blood may have a distinct protein profile essential to the homeostasis preservation of the pulmonary arteriovenous integrity^8^; this study aimed to evaluate the effects of human SVC and HV plasma on arteriovenous vascular physiology, specifically endothelial cell behavior, using 2D assays and 3D OoC in vitro models, coupled with comparative human plasma proteomic analysis. This research represents an initial step towards understanding how anatomically distinct plasma sources may modulate vascular pathophysiology, with potential relevance for future translational therapies targeting PAVM.

## Results

Per approval from the Kyoto University Ethics Committee (ID: R2560), paired full blood samples from SVC and HV were collected during routine cardiac catheterization in a prospective cohort of 10 infants with diverse cardiac congenital diseases (Table 1). Plasma was extracted and stored at -80 °C until experimental use.

**Table 1:** Patient demographic and clinical data.

Case #	Age	Sex	Diagnosis	SpO_2_ (%)	Qp/Qs	Pp/Ps	Rp
1	2y	Female	Patent ductus arteriosus	97	1.00	0.22	1.65
2	0y11m	Male	Transposition of the great arteries (Post-operation)	98	1.00	0.27	0.70
3	0y11m	Male	Transposition of the great arteries (Post-operation)	97	1.00	0.23	1.44
4	0y4m	Male	Atrial septal defect. Pulmonary hypertension Down’s syndrome	95	1.52	0.63	6.55
5	0y10m	Female	Atrial septal defect. Pulmonary hypertension	96	2.33	0.26	1.06
6	1y	Male	Coarctation of the aorta (Post-operation)	97	1.00	0.53	5.94
7	0y2m	Female	Patent ductus arteriosus Isolated right pulmonary artery	86	-	-	-
8	0y4m	Male	Ventricular septal defect	97	1.33	0.22	2.19
9	0y6m	Male	Atrial septal defect	95	3.77	0.51	1.54
10	0y4m	Male	Ventricular septal defect	97	2.90	0.61	2.60

SpO_2_: Peripheral oxygen saturation. Qp/Qs: the ratio of pulmonary blood flow (Qp) to systemic blood flow (Qs). Pp/Ps: the ratio of pulmonary systolic pressure (Pp) to systemic systolic pressure (Ps). Rp: Pulmonary arterial resistance value indexed to body surface area. Not available data (-).

### Superior Vena Cava plasma supplementation leads to increased cell migration: in vitro scratch assay

**Fig. 1:**
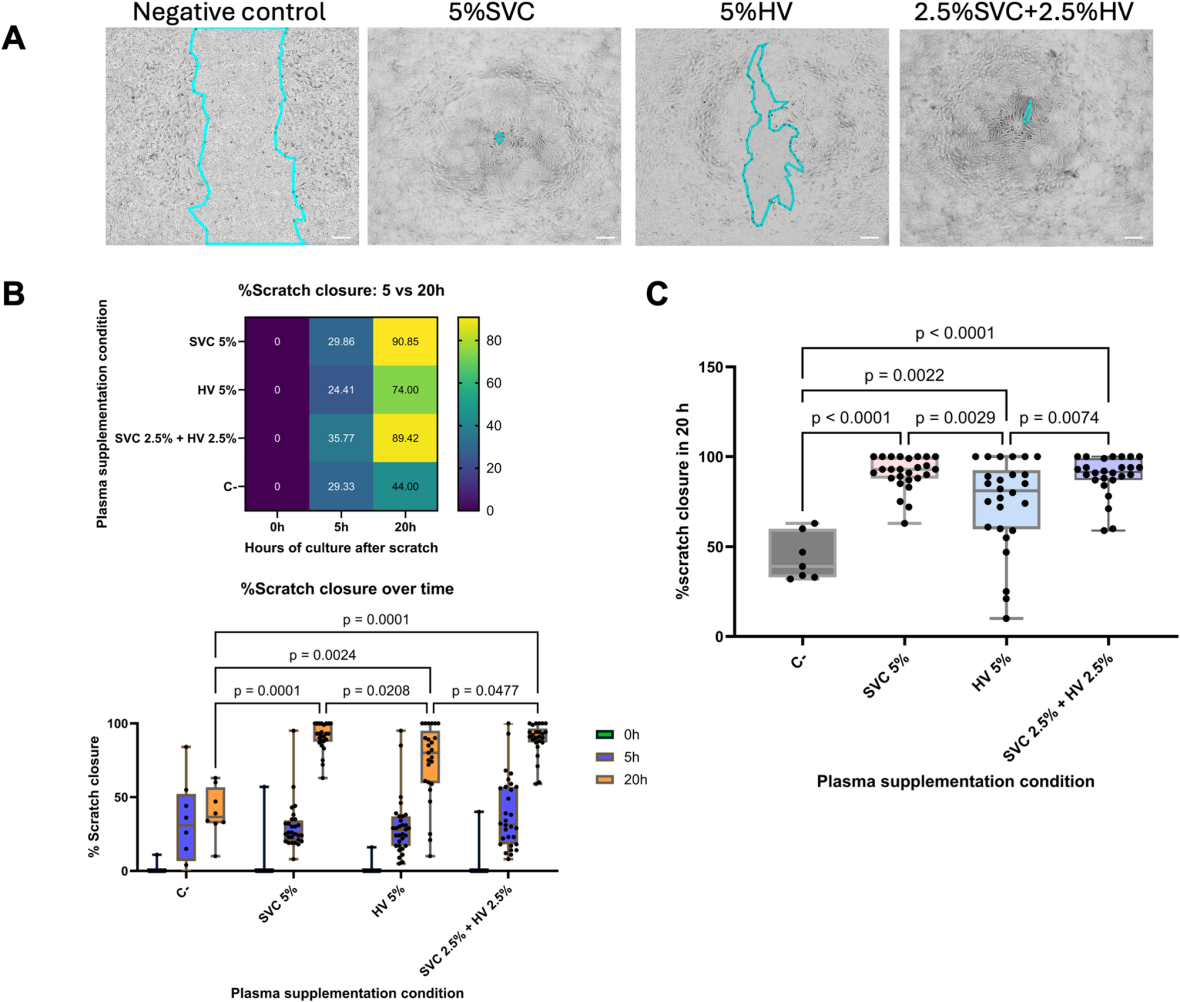
In vitro scratch assay analysis. **A**) Representative images showing scratch closure after 20 hours (h) of co-culture of endothelial and smooth muscle cells at a 5:1 seeding ratio. Scale bars: 200 μm. **B**) Top: Percentage of scratch closure at 5 and 20 h, relative to baseline at 0 h. Bottom: Multiple comparison analysis using a mixed model to assess the effect of different plasma supplementation conditions at 0, 5, and 20 h. **C**) Percentage of scratch closure at 20 h. Statistical significance between plasma supplementation conditions is indicated.

SVC and HV plasma effect on angiogenesis potential was evaluated using two methods. First, cell migration modulation potential was evaluated using endothelial (EC) and smooth muscle cell (SMC) in 2D co-culture on an in vitro scratch assay analysis (Fig. 1A). Cell migration over the scratch area was measured and compared at 0, 5 and 20 hours (h) of culture (Fig. 1B). No significant difference in the percentage of scratch closure was evidenced in the first 5 h after differential plasma exposure (Fig. 1B). After 20 h, 5% HV plasma supplemented condition showed a significantly reduced percentage of scratch closure compared to SVC (*p* = 0.003), SVC and HV combined condition (*p* = 0.007) and negative control with no plasma supplementation (*p* = 0.002) (Fig. 1A, B and C). All plasma supplemented conditions showed increased migration compared to control (*p* = 0.002), however there was no significant difference between SVC and SVC + HV plasma supplemented conditions (Fig. 1C).

### Hepatic vein a plasma supplementation leads to decreased endothelial cell sprouting and elongation: in vitro tube formation assay and validation with 3D MultiCUBE arteriovenous in vitro model

**Fig. 2:**
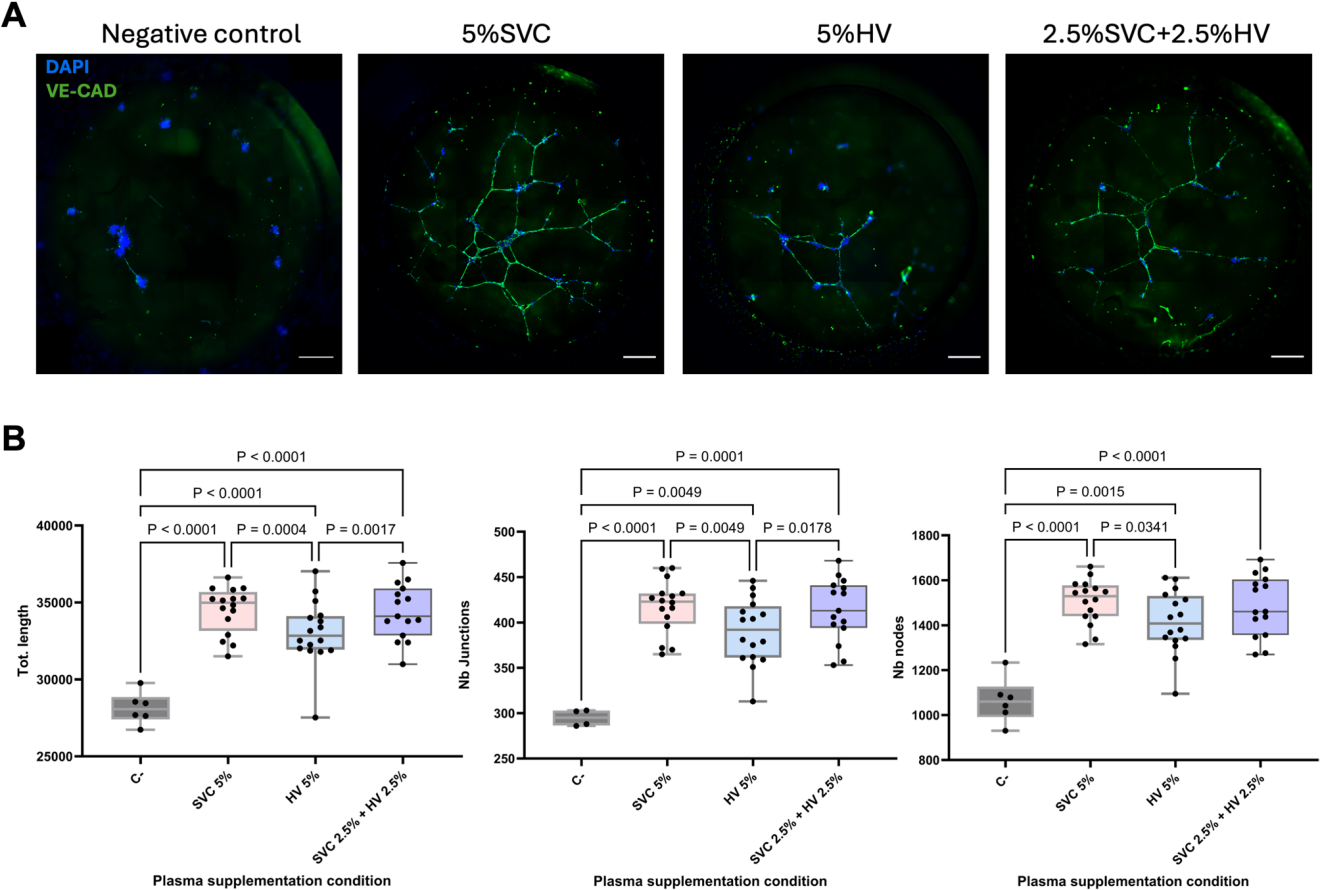
Arteriovenous tube formation assay. **A**) Representative images showing the effect of plasma supplementation from the superior vena cava (SVC), hepatic vein (HV), and a 1:1 mixture of SVC and HV (SVC + HV) compared to a negative control (no plasma) on arteriovenous endothelial tube formation after 6 hours (h) of culture. VE-CAD, VE-cadherin (green); DAPI, 4’,6-diamidino-2-phenylindole (blue). Scale bars: 500 μm. **B**) Quantification of tube formation after 6 h of culture. Tot. length, total network length; Nb junctions, number of junctions; Nb nodes, number of nodes. Superior vena cava (SVC), hepatic vein (HV), negative control (C–).

Second, angiogenic potential regulation on EC was evaluated with an in vitro tube formation assay as described in the methods. Vascular tube formation was observed in all conditions. However, increased tube formation potential was observed in all plasma supplemented conditions compared to control (Fig. 2A and B). Increased branching number (*p*<0,0001), branching interval (*p* = 0,0002) and total branching length (*p* = 0,0002) were observed in all plasma supplemented conditions (Supplementary Fig. 1A), overall creating complex tubular networks with an increased total network tube length (*p* = 0,0001). Furthermore, no significant differences were observed in the number of meshes or mesh index between plasma conditions and control (Supplementary Fig. 1B). This indicates that although there is an increased angiogenic potential of EC secondary to plasma exposure, with higher sprouting and tubular elongation, the network formation is still immature and lacks stabilization. Within plasma supplemented conditions, HV plasma only condition showed decreased sprouting with lower number of nodes (*p* = 0,034), junctions (*p* = 0,005) and limited tube elongation with a lower total tube length formation (*p* = 0,0004) compared to SVC supplemented conditions (Fig. 2B). However, no significant difference was observed between SVC and the physiological mimicking combination of SVC and HV condition (Fig. 2B).

**Fig. 3:**
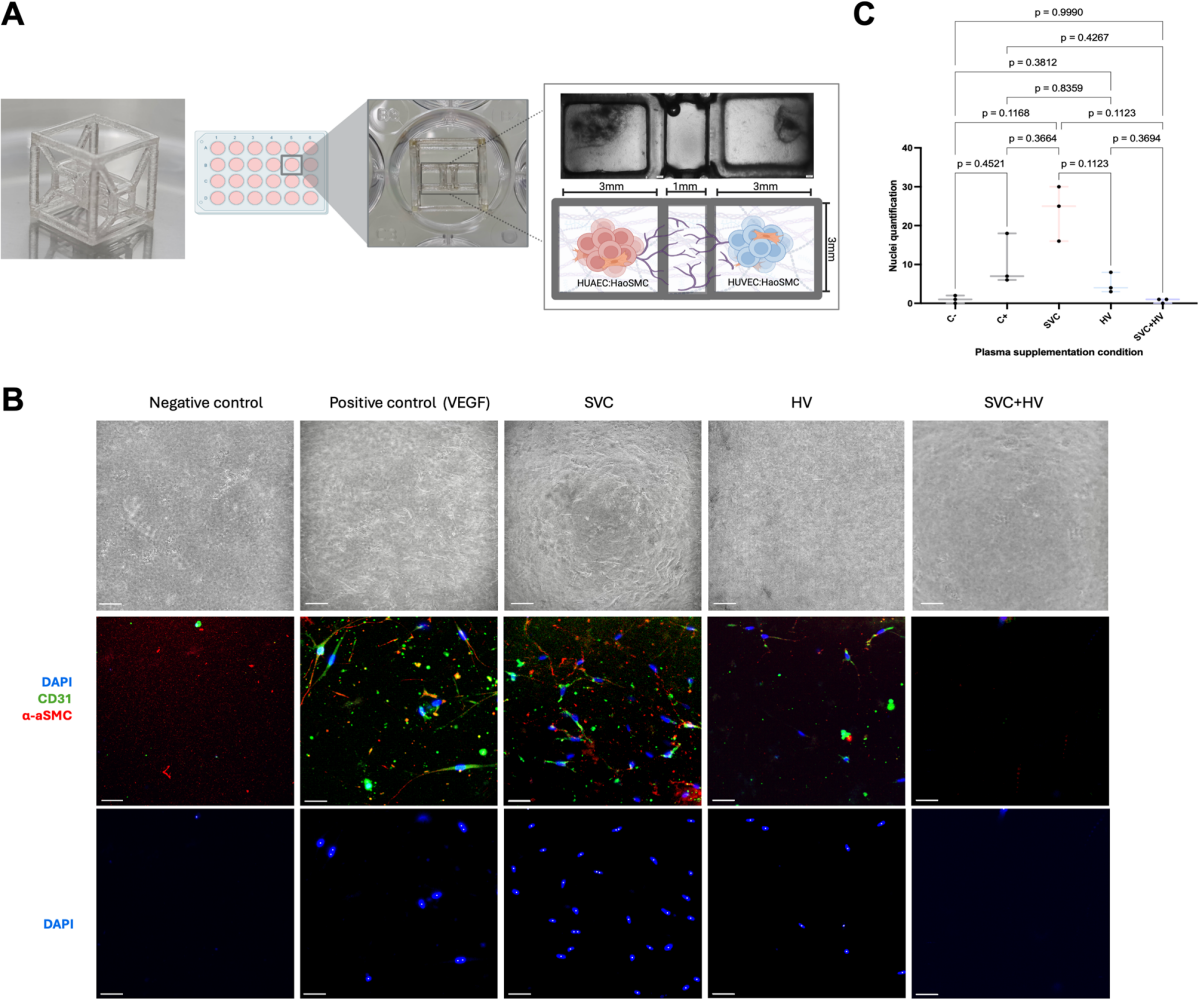
Multiple cubic unit-based scaffold MultiCUBE arteriovenous model. **A**) Schematic representation of the MultiCUBE arteriovenous model. Left: Photograph of the MultiCUBE unit design. Center: Standard placement of the MultiCUBE scaffold in a 24-well culture plate. Right (top): Bright-field image of the MultiCUBE after extracellular matrix (fibrin) assembly, showing a continuous fibrin hydrogel across all three cubes, with specific localization of cells in the lateral cubes and a central connecting cube containing only fibrin. Right (bottom): Schematic illustration of the arteriovenous model setup. **B**) Bright field and corresponding immunofluorescence images of the central cube in the MultiCUBE scaffolds after 10 days of culture under various plasma supplementation conditions. CD31 (green) to identify endothelial cells, α-aSMC (red) for smooth muscle cells, and DAPI (blue) for nuclei. The lower panel highlights differences in total observable nuclei (individual nuclei depicted by white squares) across the condition. **C**) DAPI based nuclei quantification; although the median distribution is distinct among conditions, no statistically significant differences were found between groups (p≥0.05). Bright-field image scale bar: 200 μm; immunofluorescence image scale bar: 50 μm.

Next, a multiple cubic unit-based scaffold culture system (MultiCUBE)^9^ was used to recapitulate the 3D physiological structure of arteriovenous connection development (Fig. 3A). Supplementation of differential anatomical origin plasma on each MultiCUBE culture media showed apparent variations of the angiogenic potential induction per condition; however, none of the comparisons achieved statistical significance (*p*≥0.05). The analysis of median (50% percentile) distribution across conditions, although statistically non-significant, revealed a tendency towards increased, sprouting, elongation and migration of EC and SMC in SVC supplemented cultures; exceeding even the positive control which was the only condition supplemented with vascular endothelial growth factor (VEGF) (*p* = 0.3664) (Fig. 3B and C). Furthermore, cells under SVC condition exhibited strong co-expression of alfa actinin smooth muscle and pan-endothelial CD31 marker. In contrast, HV plasma exposed MultiCUBE evidenced limited sprouting and migration of cells, with almost no elongation compared to SVC, though again this difference was not statistically significant (*p* = 0.1123) (Fig. 3B and C). The combination of SVC and HV plasma, mimicking normal circulation, yielded lower median sprouting and migration values than HV alone, comparable to the negative control without plasma or VEGF, with no statistically significant differences observed (*p*≥0.05) (Fig. 3B and C).

### Differential identification of low molecular weight enriched areas in the hepatic vein derived plasma protein profile

**Fig. 4:**
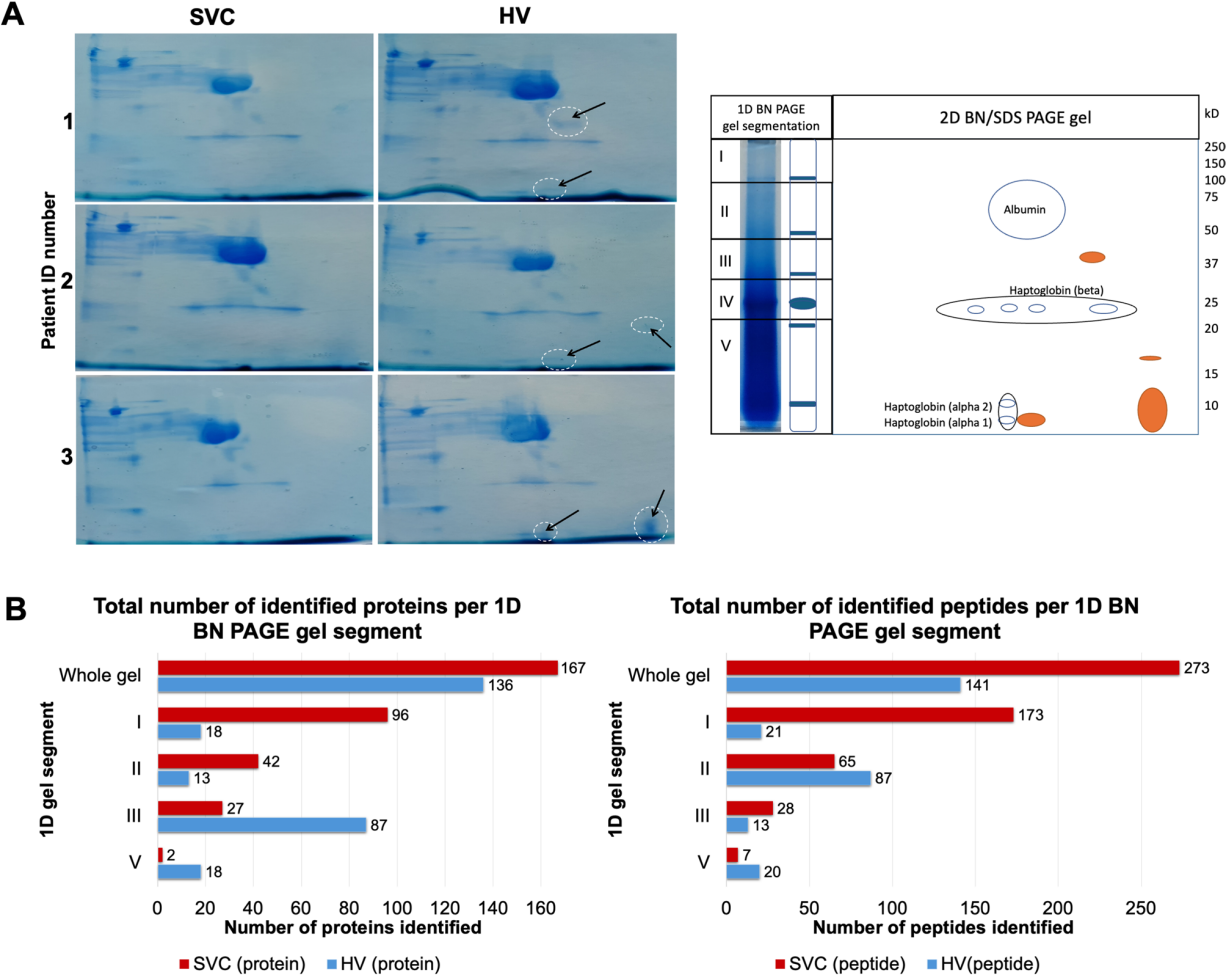
Two-dimensional Blue Native (BN) /SDS-PAGE gel visualization of protein composition differences between plasma samples from distinct anatomical origin. **A**) Representative 2D BN/SDS-PAGE gel analysis of plasma samples from the superior vena cava (SVC) and hepatic vein (HV) of three patients (Patient IDs 1–3) (left). All the gel plots from the 2D BN/SDS-PAGE for each experiment are shown. A schematic of the 2D BN/SDS-PAGE setup with corresponding 1D BN-PAGE gel segments is shown (right). Black arrows with white dotted circles (left) highlight differences in protein patterns, particularly in the low molecular weight region (< 20 kDa) of HV plasma, corresponding to segments III and V on the 1D BN-PAGE gel (right). Orange circles (right) schematically summarize the observed proteomic differences and their correspondence with the 1D gel segments. **B**) Proteomic profiling of individual 1D BN-PAGE segments (I, II, III, and V), showing the total number of proteins (left) and peptides (right) identified per segment.

Since the preceding results demonstrated that HV plasma suppresses EC migration, we next analyzed the proteins potentially mediating this effect. As a first approach we implemented two-dimensional blue native/ sodium dodecyl sulfate polyacrylamide gel electrophoresis (2D BN/SDS-PAGE) method, to characterize multi-protein complexes in our human plasma samples^10^. For example, haptoglobin (Hp), an “acute phase” protein, has different functions depending on its genetic polymorphism (1–1, 2 − 1, and 2–2)^11^. Through 2D BN/SDS-PAGE plasma proteomic pattern analysis, Hp genetic polymorphisms were distinguished, and our subjects were clearly diagnosed as Hp1-2 (Fig. 4A).

By comparing the protein complexes composition separation profiles of plasma per origin (SVC and HV), discrepancies in the 2D SDS-PAGE protein pattern were evident and especially prominent in the lower molecular weight areas of the HV plasma samples 2D gel, suggesting the presence of differentially expressed smaller molecular size proteins between SVC and HV derived plasma. (Fig. 4A). These findings were further supported by liquid chromatography-tandem mass spectrometry analysis (LC-MS/MS) protein identification profiles on 1D BNPAGE gel samples. Differential proteomic profiles were evidenced according to plasma origin. 273 peptides and 167 proteins were detected in SVC. Meanwhile 141 peptides and 136 proteins were retrieved in HV plasma sample. Analysis of 2D BN/SDS-PAGE proteomic patterns based on correspondence with 1D BN-PAGE gel segments (Fig. 4A and B), indicated that SVC, has a prominent composition of higher molecular weight protein areas of the 1D gel, while hepatic vein proteome complexity was found to be rather prominent in the lower molecular weight areas (Fig. 4B).

### SVC plasma proteome has a pro angiogenic profile, while HV plasma proteome relates to cell adhesion complex enhancement

**Fig. 5:**
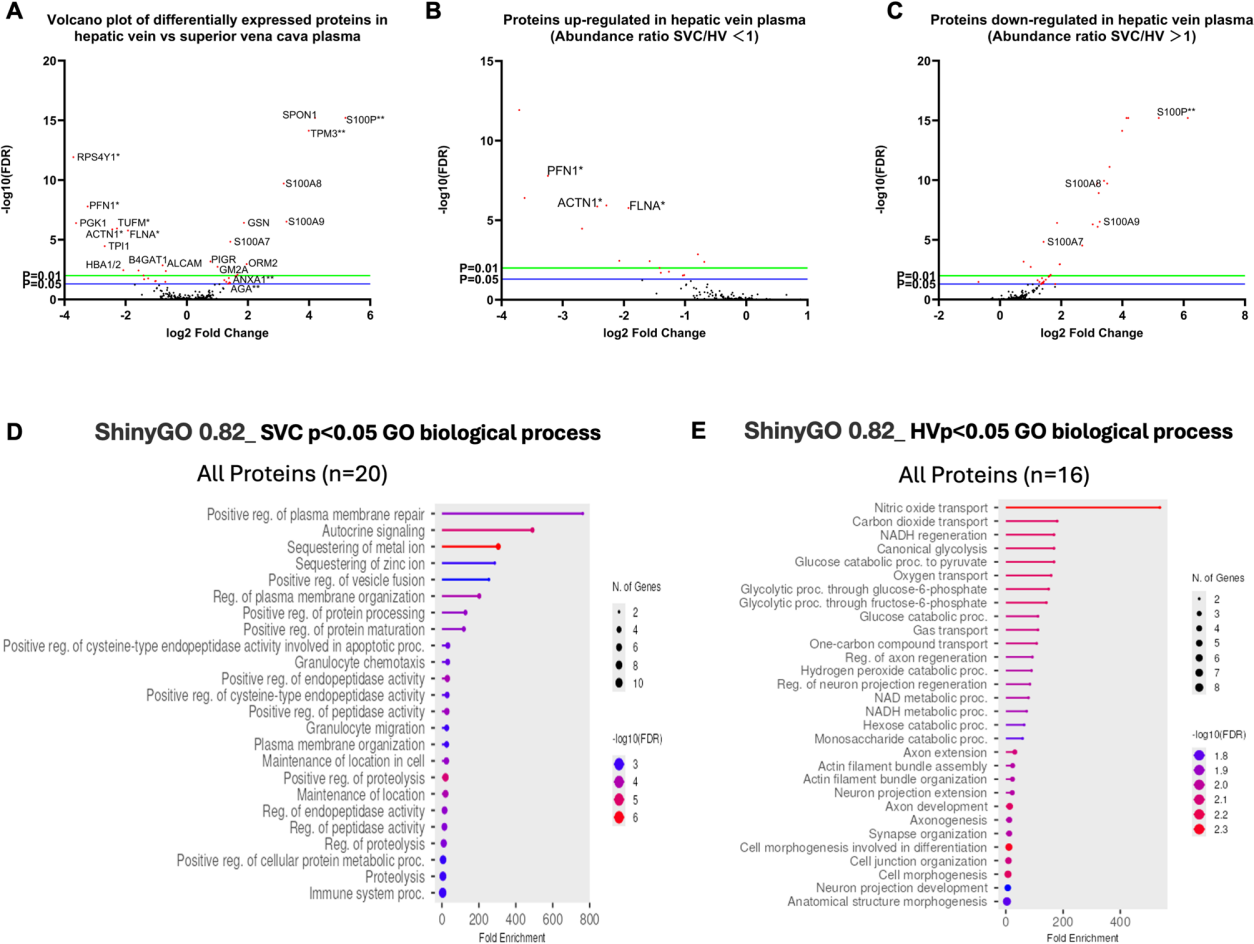
Differences between SVC and HV human plasma proteome profiles. **A**) Volcano plot illustrating up and downregulation proteins in hepatic vein plasma compared to superior vena cava. A total of 364 proteins had differential expressions between SVC and HV plasma samples (*p* < 0.05). Proteins uniquely expressed in HV are marked with an asterisk (*), and those uniquely expressed in SVC are marked with a double asterisk (**). **B**) Upregulated proteins in HV plasma with known vascular physiological relevance. Uniquely expressed HV proteins are indicated with an asterisk (*). **C**) Downregulated proteins in HV plasma (preferentially expressed in SVC plasma) with vascular physiological relevance. Uniquely expressed SVC proteins are indicated (**). Statistical significance thresholds were defined using –log₁₀ (False discovery rate (FDR)):–log₁₀(FDR) ≥ 1.3 corresponds to FDR-adjusted p-value < 0.05, –log₁₀(FDR) ≥ 2.0 corresponds to FDR-adjusted p-value < 0.01. **D**) Functional gene ontology biological process analysis including of all upregulated proteins in superior vena cava (SVC). E) Functional gene ontology analysis including of all upregulated proteins in hepatic vein (HV). D) and E) enrichment analyses were conducted using ShinyGO v0.82.

Given the low number of proteins identified in the previous approach, we speculated high abundant proteins could be interfering with lower molecular weight protein detection. Optimization of sample preparation for proteomics was performed using high abundant protein depletion columns coupled with protein clean up and elution using single pot solid phase enhanced sample preparation method as detailed in the methods, using patient ID number 3 samples. LC-MS/MS analysis retrieved a total of 5658 peptides corresponding to 701 identified proteins, excluding contaminants. During data processing, 125 missing values, 210 proteins identified with ≤ 1 unique peptide, and 2 proteins with an abundance ratio (SVC/HV) = 1 were filtered out, resulting in 364 proteins with differential expression between SVC and HV samples of which only 36 proteins were statistically significant (*p* <0.05) (Fig. 5A and Supplementary Table 1). Sixteen proteins were found to be upregulated in HV sample (abundance ratio [SVC/HV] < 1), five of which were uniquely expressed in HV plasma (Fig. 5A and B). On the other hand, there were 20 downregulated proteins in HV (abundance ratio [SVC/HV]>1); of these, 4 proteins were unique to SVC, while the resting 16 had a predominant expression in SVC plasma rather than HV (Fig. 5A and C).

SVC plasma proteome showed significant enrichment of gene ontology (GO) functional pathways related with positive plasma membrane repair and organization (GO biological function -log10(FDR) = 4.0) (Fig. 5D). Toll-like receptor 4 binding (TLR4) was the top enriched pathway (GO molecular function -log10(FDR) = 4.0), along with other like RAGE receptor binding, previously reported to be related with promotion of angiogenesis (Supplementary Fig. 2). KEGG pathways showed enhancement of IL-17 signaling pathway (KEGG -log10(FDR) = 4.0) (Supplementary Fig. 2). Furthermore, although non statistically significant, it is worth mentioning that functional gene ontology analysis of the proteins found to be uniquely expressed in SVC plasma indicated modest enrichment of endothelial and epithelial cell migration (-log10(FDR) = 1,27) (Supplementary Fig. 2). In parallel, uniquely expressed proteins in SVC showed a significant enrichment on pathways related to glycan degradation (KEGG -log10(FDR) = 1.58).

On the other hand, HV derived plasma proteomics indicated a significant enrichment of cell to cell junction pathways (DAVID GOTERM_CC *p* < 0,009), actin filament binding (DAVID GOTERM_CC *p* < 0,015), focal adhesion (DAVID KEGG *p* < 0,034; GO biological function (-log10(FDR) = 2,25)) adherence junction (GO biological function -log10(FDR) = 2,25), cell adhesion molecules (GO biological function -log10(FDR) = 2,00), actin binding (DAVID GOTERM_MF *p* < 0,031; GO biological function (-log10(FDR) = 2,00) and cytoskeleton organization clusters (DAVID KEGG *p* < 0,43) related to the 16 HV upregulated expression genes (Fig. 5E and Supplementary Fig. 3). For the 5 proteins uniquely found in HV samples, focal adhesion and regulation of actin cytoskeleton pathways were enriched through KEGG pathways analysis (Supplementary Fig. 3), while gene ontology biological functional analysis showed concomitant enrichment of actin filament organization and assembly (GO biological function -log10(FDR) = 2,50), actin cytoskeleton (GO biological function -log10(FDR) = 1,50) and cell junction organization (GO biological function -log10(FDR) = 2,00) reinforcing the findings on the upregulated protein group analysis. (Supplementary Fig. 3).

## Discussion

PAVM pathogenesis has been associated with disrupted angiogenic balance in pulmonary vasculature after SCPC due to loss of “hepatic factor” control^5,12^. Prior animal studies support this hypothesis. Starnes et al. demonstrated increased microvessel density with histological features resembling PAVMs in an SCPC rat model^13^; while Kavarana et al. observed elevated proangiogenic markers and endothelial proliferation in a porcine SCPC model within six weeks^12^. The 2D arteriovenous in vitro models used in this study further validated these findings. Both scratch and tube formation assay data showed enhanced endothelial sprouting and elongation with SVC plasma (Figs. 1A and C and 2A and B). However, mesh network formation remained unchanged (Supplementary Fig. 1B), likely due to the absence of supporting pericytes^14^ and inherent limitations of the 2D assay design. Furthermore, interestingly our findings differ from those of Spearman et al., despite similar in vitro approaches^15^. Nevertheless, differences in cell types, plasma vs. serum use, and handling conditions, particularly if the hepatic factor is labile, might explain these discrepancies. Given the limitations of 2D systems to reproduce complex biological processes, we proceeded to assess these effects in a more physiologically relevant model.

The pro-angiogenic profile of SVC plasma was confirmed using the 3D MultiCUBE arteriovenous model, showing higher angiogenic activity compared to both control and HV plasma. However, co-supplementation of SVC with HV plasma resulted in limited angiogenesis, comparable to the negative control and even lower than HV plasma alone, although differences were not statistically significant (Fig. 3B and C). Nevertheless, this aligns with physiological expectations, as SVC and HV blood normally converge in pulmonary circulation, where balanced modulation likely limits aberrant angiogenesis. The extracellular matrix (ECM), a key regulator of endothelial behavior, has recently emerged as a critical factor in single ventricle pathology^16^. The MultiCUBE model not only enables 3D angiogenic growth but also provides an ECM-rich environment that mimics more closely arteriovenous development in vivo compared to 2D systems, which may explain the differences observed across models. This is the first application of an arteriovenous 3D OoC to study of PAVM physiology, allowing to discern human plasma modulation over angiogenic potential per anatomical origin. Nonetheless, further validation of these observations is required, given the limited sample number in our study.

In vitro findings of SVC angiogenic potential were further supported by the plasma proteome analysis. S100 calcium-binding protein A7 (S100A7), A8 (S100A8), A9 (S100A9) and P (S100P), known for their roles in angiogenesis and immunomodulation^17^, were found significantly downregulated in HV compared to SVC plasma (Fig. 5A and C). S100 family proteins can be detected in plasma and have specific binding with transmembrane protein TLR4, the most highly expressed toll like receptor modulating endothelial immune response and cell barrier integrity regulator^18^. Interestingly, TLR4 was also the top enriched GO molecular pathway associated to SVC upregulated proteome. S100A7 enhancement has been observed in early stages of tumor progression, as well as angiogenesis promotion by the transmembrane receptor for advanced glycation end products (RAGE) binding, evidenced on previous HUVEC studies^19^, as well as here (Supplementary Fig. 2). S100A8, along with its heterodimer S100A9 have been widely associated with endothelial dysfunction and even considered as a biomarker for vascular diseases^20^. Wang et al., described that S100A8 binding to TLR4 on EC induces MAPK signaling pathway and leads to acute disarrangement of F-actin and ZO-1 tight junction organization in HUVEC, resulting in increased permeability and disruption of the endothelial cell barrier as early as 30 minutes after exposure in vitro^21^.

Furthermore, SVC proteome significant enrichment of pathways related to cell membrane regulation in inflammatory modulation responses like positive plasma membrane repair and organization, enhancement of IL-17 signaling pathway, glycan degradation and although non statistically significant, EC migration, are all associated to S100 family protein function (Fig. 5D and Supplementary Fig. 2). These results suggest that SVC plasma may promote EC activation, increased membrane permeability and angiogenesis through upregulation of S100 family derived proteins; explaining the increased migration, sprouting and elongation of vessels in all our in vitro models with SVC plasma supplementation. Nevertheless, further studies that validate the reproducibility of the proteomic profile from SVC plasma are required, as there is enhanced variability between reports regarding SVC proteome profile^22,23^.

Moreover, vascular physiology extends beyond the endothelium. PAVM compromise not just capillaries, but higher vascular hierarchical structures like precapillary, conduit and branching vessels^24^. Animal and human studies have associated the role of vascular tone modulators with PAVM pathogenesis after SCPC. Malhotra et al., unilateral Glenn lamb model proposed PAVM association with angiotensin II downregulation leading to altered endothelial vascular tone^25^; while Duncan et al. suggested that increased intracellular junctions between EC may play a role, along with abnormal angiogenesis, in the development of PAVM^24^. Therefore, dysregulation mechanisms concerning not only EC but also SMC modulation over vascular tone and its interaction with the ECM as a possible mechanism leading to PAVM development requires thorough consideration.

Our HV plasma proteome showed upregulation of actin binding proteins filamin A (FLNA), profilin 1 (PFN1) and alpha actin 1 (ACTN1) (Fig. 5B); with enrichment of pathways related to cell junctions, focal adhesion, and cytoskeleton regulation (Fig. 5E and Supplementary Fig. 3). Though cytoplasmic, these proteins circulate in plasma under certain conditions^26^. Tuning of these cell adhesion complexes allows mechano-transduction regulation and interaction between EC, SMC and basal membrane in precapillary vessels^27^. FLNA deficiency leads to abnormal vasodilation and disorganized vasculature^28^ like PAVM; while PFN1 interaction with alpha-actin is crucial for regulating focal adhesion and vascular smooth muscle contractility through actin binding^29^.

These results suggest SVC proteome has the potential to enhance EC migration, which under physiological normal conditions may be counter regulated by HV plasma induced enforcement of cell-to-cell junction regulation, achieving vascular integrity conservation^30^. After SCPC, loss of the HV counterregulatory potential may upregulate SVC effects, leading to increased membrane permeability and EC migration; possibly through mechanisms like TLR4 and RAGE activation, as evidenced in this study (Supplementary Fig. 2) and previously reported elsewhere^18^. Alternatively, since our plasma samples were retrieved from non-healthy patients, it could be that SVC plasma proteome cell migration and repair induction capacity may only be triggered in pathological conditions, as part of a systemic inflammatory response. This may lead to elevation of S100 binding proteins in SVC plasma and activation of TLR4 response which has been associated with EC dysfunction, increased permeability, cell migration and potentially vessel sprouting^31^. In patients with intact hepatopulmonary circulation, this effect would be homeostatically countered by HV plasma enhancement of cell-to-cell junction adherence, preserving vascular integrity and preventing abnormal angiogenesis. This is consistent with the enrichment of membrane integrity pathways found in the HV proteome (Fig. 5E and Supplementary Fig. 3) and supported by the conserved cell migration pattern observed in the SVC and HV plasma co-exposed MultiCUBE experiment (Fig. 3B and C).

In conclusion, this report may serve as a first step to understand PAVM development not only as a pro-angiogenic mediated event, but rather a dysregulation of cell adhesion complex control over vascular tone regulation and ECM interplay. Nevertheless, our study presented multiple limitations of important consideration. First, none of the included patients had univentricular heart disease due to limited case availability at our institution. Further studies including single ventricle patients are essential, as our findings may not translate to this population. Notably, our cohort had elevated pulmonary-to-systemic blood flow ratios (Qp: Qs), which differ from typical post-Glenn physiology, further highlighting the need to validate these results in single ventricle patients. Second, our findings reflect physiological differences between SVC and HV plasma in vascular physiology, not PAVM pathological states, as none of the included patients had SCPC or any type of hepatopulmonary interruption. Third, the overall sample size, specifically in the 3D MultiCUBE experiments is limited, and further replicate validation is required. Fourth, HUVECs and HUAECs were used due to their well-known reproducibility, wide validation in angiogenesis studies, and established use in plasma-stimulated assays, as well as their availability at our institution. However, future studies incorporating pulmonary-specific arterial and venous EC are needed to develop more anatomically relevant models^32,33^. Fifth, the total number of identified proteins were lower than expected for high complexity samples such as human plasma, with observed variability in proteome profiles depending on the analytical method used even with the same sample. The huge dynamic range of plasma components, posttranslational modifications, degradation mechanisms and protein loss during sample preparation may account for the discrepancies among reports that have tried to unveil the “hepatic factor” using SCPC plasma proteome profiling^22,23^, including ours. Studies that can confidently account for interpatient variability and differences between proteomic methodologies are required.

Finally, we hope that our use of proteomics technology in the evaluation of clinically relevant samples coupled with the development of an in vitro physiologically relevant 3D arteriovenous model may serve as a new tool to facilitate future efforts in the understanding of PAVM pathophysiological mechanisms and the generation of translational therapeutic alternatives that can directly improve patient outcomes in the near future.

## Methods

### Study population and collection of human plasma samples

This study was approved by the Ethics Committee of Kyoto University (ID: R2560) and conducted in accordance with the principles of the Declaration of Helsinki. Written informed consent was obtained from all participants, or from their legal guardians when applicable. All data used in this study were pseudonymized prior to analysis and publication. The data was handled in such a way that no individual could be identified directly or indirectly.

Paired full blood samples were collected from SVC and HV just before the drainage to the inferior vena cava (IVC), during routine catheterization of patients with diverse cardiac congenital malformations, without PAVM, from 0 to 2 years of age (*n* = 10). Patients undergoing urgent cardiac catheterization or hemodynamically unstable patients were excluded from the study. Informed written consent as described above was obtained to undergo cardiac catheterization, plasma sample collection and use of clinical data as participants of the current research.

Within 2 h of whole blood collection, plasma was extracted by centrifugation at 2,000 gravitational units (g) for 10 min in a refrigerated centrifuge (4 °C). Following centrifugation, the supernatant (plasma) was transferred into sterile polypropylene tube (1.5 ml Eppendorf tubes) in 100ul aliquots to avoid thaw and refreeze cycles; labeled and stored at -80 °C until experimental use.

### In vitro scratch assay

A cell suspension (2 × 10^4^ cells/ml) composed of venous and arterial endothelial cells (HUVEC + HUAEC) (PromoCell, Cat. No C-12200 and C-12202 respectively, Heidelberg, Germany) along with human aortic smooth muscle cells (HAoSMC) (PromoCell, Cat. No C-12533) were seeded in a 5:1 ratio (HUVEC + HUAEC: HAoSMC) on a 96 well flat bottom plate and cultured in control plasma free Endothelial Cell Growth Medium 2 (EGM-2; PromoCell, Cat. No. C-22011) until 90–100% confluent. Then, a straight vertical midline scratch was performed on each well using 200 µl pipette tips, followed by supplementation with four different conditioned medium (EGM-2 for negative control/ and EGM + plasma (5%SVCplasma/ 5%HVplasma/ 2.5%SVC + 2.5%HV)). All conditions were supplemented with VEGF 50ng/ml each. Phase contrast images were obtained at 0, 5, 20 h of incubation and posteriorly analyzed using Wound_healing_size_tool plugin in ImageJ calculating the percentage of wound closure per timepoint as previously published^34^.

### Tube formation assay

A tube network formation potential assay was performed on Matrigel (Corning Life Sciences, Cat No. 356231, Corning, NY, USA) precoated µ-Slide 15 Well 3D Glass Bottom well (Ibidi µ-Slide Angiogenesis, Cat. No. 81507. Gräfelfing, Germany). A combination of HUVEC and HUAEC (1:1 ratio) were used to mimic the continuum arteriovenous phenotype on the pulmonary capillaries passages no. 2–4. Cells were thawed and passaged once in EGM-2 before being dissociated and collected for experiment use at 80% confluency state. On the day of cell dissociation, 10 µl of previously thawed batch of Matrigel (overnight at 4 °C) were used to precoat every 3D µ-Slide well as per manufacturer protocol and incubated at 37 °C for 1 h. A cell suspension of 2 × 10^5^ cells/ml of HUVEC: HUAEC (1:1 ratio) was achieved to seed 10,000cells/well in 50ul of EGM-2 per well. 5 different conditions of medium were used for culture at 37 °C, 5%CO2. Negative control (EGM-2 only), HV (5% HV derived plasma in EGM-2), SVC (5% SVC derived plasma in EGM-2) or SVC + HV (2,5% HV + 2,5%SVC plasma in EGM-2), no further supplementation was used. Phase contrast images were obtained at 0, 1, 6 and 24 h of incubation and posteriorly analyzed using Angiogenesis Analyzer plugin for Image J software^35^.

### MultiCUBE

A multiple-hydrogel localization cubic unit-based scaffold (MultiCUBE) culture system was used to evaluate EC: SMC co-culture self-organization and vascular formation capacity into specific locations on the hydrogel following a previously reported modified protocol^9^. In brief, fibrin hydrogel (Fibrinogen 5 mg/ml (Sigma-Aldrich, Cat No. F8630, St. Louis, MO, USA) + thrombin 50U/ml (Sigma-Aldrich, Cat. No. T4648)) was localized in all 3 parallel cubit units. HUAEC and HAoSMC (5:1 ratio) along with HUVEC and HAoSMC (5:1 ratio) were seeded separately in the lateral cube units, making sure the center unit was left untouched, containing only fibrin hydrogel to allow vascular growth and arteriovenous network connection formation evaluation afterwards. After 20 min incubation at 37 °C, prewarmed Endothelial Growth Medium (EGM; PromoCell Cat. No. C-22010) was added to all conditions along with differential plasma supplementation to each MultiCUBE. Five different conditions were set (positive control (EGM-2 + VEGF 50ng/ml), negative control (EGM only), HV (5% HV derived plasma in EGM-2), SVC (5% SVC derived plasma in EGM-2) or SVC + HV (2,5% HV + 2,5%SVC plasma in EGM-2). Medium change was performed every other day. MultiCUBES were fixed at day 10 of culture, stained and evaluated using fluorescence microscopy imaging and analysis of vascular network formation in the ECM central cube region per plasma condition. Cell number in the center ECM cubic unit was evaluated by nuclei quantification using the Spot detection tool in Imaris, version 10.1 (Bitplane, an Oxford Instruments Company, Zurich, Switzerland) on maximum intensity projections of DAPI-stained images. Prior to analysis, Z-stack size was normalized across all samples to ensure consistency in projection depth and quantification accuracy. Illustration of the MultiCUBE (Fig. 4A) was created with BioRender (BioRender.com, BioRender Inc., Toronto, Canada).

### BN 2D SDS PAGE electrophoresis separation of human plasma sample protein components

Blood plasma was used to perform proteome multicomplex analysis using 2D BN/SDS PAGE separation technique for all the selected samples slightly modifying a previously described method^36^. Briefly, 3µL of plasma, 300µL of blue native buffer (6-aminohexanoic acid, 200 mM Bistris, 500 mM EDTA, 5 M NaCl, and 20% glycerol) and protease inhibitor were centrifuged at 4 °C 15,000 rpm for 2.5 h on a 10 kDa centrifugal filter (Amicon UFC201024). After centrifugation, the residual protein complexes remaining on the filter were collected on a new microtube with a quick centrifugal flush, obtaining around 3µL per sample. BNB based dye (coomassie 250 g and 750 mM aminocaproic acid: 10% of the sample volume) was then added to charge and ionize the protein complexes collected, preparing them for the first-dimension blue native polyacrylamide gel electrophoresis in gel (BN-PAGE). 1D BN-PAGE was done in parallel for all the samples as follows. Two 4–15% polyacrylamide gels (Mini-PROTEAN^®^ TGX Precast Gels; Bio-Rad, Hercules, CA, USA) were soaked in 1D running buffer (25 mM Tris and 192 mM glycine) and set in the electrode chamber into the electrophoresis tank filled with the running buffer. 10µL of the BNB dyed samples were aliquoted per lane of the gel. The source of energy was set at 50 V and the electrophoresis equipment was left under room temperature conditions for 1 h. Afterwards, the voltage was increase to 90 V until 300 min had past or the sample had completed running through the gel. The gels were then soaked in 100 mL of reducing solution (dithiothreitol 1%, Tris HCl 0,25 M, glycerol 20% ) for 30 min. One of the gels continued to second dimension (2D) SDS PAGE and the other one was stored at 4 °C in reducing solution for future shotgun proteomics analysis.

For the 2D SDS-PAGE, the 1D BN-PAGE gel was cut along the well edges and fed individually into a separate 12% gel lane (Mini-PROTEAN^®^ TGX™ Precast Protein Gels, 7 cm IPG/prep well; Bio-Rad Laboratories, Hercules, CA, USA.) and standard dye (Precision Plus Protein™ Dual Color Standards, Cat. No. 1610374; Bio-Rad Laboratories) was added on the edge of the gel as a control and molecular weight indicator parameter. Running buffer (25 mM Tris, 192 mM glycine, and 0.1% (w/v) SDS) was added into the electrophoresis chamber and the source of energy was set at 100 V for 100 min at room temperature. Posteriorly the gels were stained with Coomassie Brilliant Blue dye (CBB R-250; Sigma-Aldrich, Cat. No. 27816, St. Louis, MO, USA) for 60 min on shaker to make the proteins visible. The final staining pattern was recorded and compared.

### Proteomics sample preparation and proteomic analysis

#### 2D BN/SDS-PAGE gel coupled proteomics

In preparation for LC MS/MS, 1D BN-PAGE gels were cut into 5 segments (I to V) per molecular weight range (Fig. 4A). Using the previously obtained 2D gel staining pattern as a reference, 1D gel segment selection for shotgun proteomics was planned. To enhance the detection of smaller, lower molecular weight proteins by the LC MS/MS, high molecular weight abundant protein (e.g. albumin) containing segments (segment IV) of the 1D gel were excluded from the in-gel digestion. Selected gel segments were then rinsed, shrunk with acetonitrile (ACN) and then vacuum centrifuge for 2 h until dry. Then samples were incubated at 56 °C for 1 h in a reducing solution (1 M dithiothreitol + 100mM NH_4_HCO_3_), followed by alkylation in iodoacetamide (IAA) solution at room temperature, with light protection and on shaker for 45 min. After discarding IAA, the samples were washed with ammonium bicarbonate on shaker for 10 min (100mM NH_4_HCO_3_) and then ACN for 5 min, twice. Finally, samples were dried with vacuum centrifuge for 2 h and then enzymatically digested with 20ng/µL trypsin solution, on ice for 45 min, making sure the gels were completely covered. Afterwards, the samples were incubated at 37 °C for 3–4 h and centrifuged at 12,000 rpm for 1 min at room temp. The resulting solution was collected into a separate 1.5 ml tube (solution A) and the remaining gel segment was mixed with ammonium bicarbonate (20mM NH_4_HCO_3_ ) and shook for 10 min at room temperature. The solution obtained from the gels with NH_4_HCO_3_ (solution B) was collected and mixed with solution A. Shotgun proteomics of the resulting solution was performed on timsTOF Pro with nanoElute LC system (Bruker Daltonik GmbH, Bremen, Germany). Protein annotation was done withProteinScape4.0 (Bruker Daltonik GmbH).

#### Human plasma sample proteomics

Plasma samples were thawed on ice and high abundant proteins were depleted using high abundant protein depletion columns (High-Select™ Top14 Abundant Protein Depletion Resin. Thermo Fisher Scientific, Cat. No. A36370, Waltham, MA, USA) as per manufacturer protocol. The resulting enriched low-abundance protein plasma was washed and eluted using single-pot solid-phase-enhanced sample preparation method following a previous report^37^. The sample was then trypsinized (MS-Grade Trypsin/Lys-C Mix; Thermo Scientific, Cat. No. 90057) and coupled with label-free data-dependent acquisition liquid chromatography-tandem mass spectrometry (LC-MS/MS). LC-MS/MS was performed using an Orbitrap Eclipse (Thermo Fisher Scientific) coupled to a Vanquish nanoflow UHPLC. Peptides were loaded onto a C18 trap column (0.3 × 5 mm, 5 μm) and separated on a C18 analytical column (0.075 × 250 mm, 1.7 μm, IonOpticks) at a flow rate of 300 nL/min. Peptides were separated using a multistep gradient with total run time ~ 195 min. MS data were acquired in data-dependent mode with a 3-second cycle. MS1 scans (375–1500 m/z) were collected at 60,000 resolution. Precursors with intensity greater than 20,000 and charge states 2 to 7 were selected for fragmentation (isolation width: 1.6 m/z; HCD energy: 30%), and fragment ions were measured at 15,000 resolution. Raw data were searched against the Homo sapiens UniProt database (UP000005640, downloaded 2024-03-04) and the cRAP contaminant database using Proteome Discoverer 2.5 with MASCOT 2.8. Two missed cleavages were allowed. Carbamidomethylation (C) was fixed; oxidation (M) and N- terminal acetylation were variable modifications. Proteins with an FDR adjusted p-value < 0.05 were considered as significant.

Subgroup differential expression analysis by abundance ratio (SVC/HV) (Adj. P-Value < 0,05) was used to identify differentially regulated proteins between SVC and HV plasma proteome profiles, as well as uniquely expressed proteins per plasma origin. Proteins with only one unique peptide identifier were not considered for analysis, along with missing values. Gene ontology biological analysis and functional pathways analysis were performed using DAVID Bioinformatics Resources 6.8 (DAVID, National Institute of Allergy and Infectious Diseases (NIAID), National Institutes of Health (NIH), USA), and ShinyGO 0.82 (ShinyGO, South Dakota State University, South Dakota, USA) platforms^38,39^.

### Immunofluorescence staining

The process followed was the same as describe before with slight modifications^9^. In brief, all samples were rinsed with DPBS and fixed with 4% paraformaldehyde phosphate buffer solution (Nacalai Tesque Inc, Cat. No. 09154-56, Kyoto, Japan) for 30 min at room temperature. Samples were washed three times for 10 min each with DPBS and stored at 4 °C until staining. For staining, samples were permeabilized with 0.5% Triton-X for 20 min at room temperature, followed by three 10 min washes with100mM glycine in DPBS at room temperature. For non-specific binding prevention, samples were blocked with Blocking One (Nacalai Tesque Inc, Cat. No. 03953-66) for 30 min at room temperature. This was followed by further blocking in 1% donkey serum in IF solution (0.2% Triton-X + 0.1% BSA + 0.05% Tween20 in DPBS), for 30 min. Tube formation assay samples were stained with primary antibody goat anti VE-Cad (1:100; Abcam, ab33168, Cambridge, UK), rabbit anti SOX17 (1:100; Invitrogen, Cat. No MA5-24885, Waltham, MA, USA), mouse anti COUP TFII (1:100; R&D systems Cat No. PP-H7147-00, Minneapolis, MN, USA). MultiCUBE was stained with primary antibody goat anti alpha-smooth muscle actin (1:200; Abcam Cat No. ab21027), rabbit anti SOX17 (1:100; Invitrogen, Cat No. MA5-24885) and mouse anti-CD31 (1:50; Dako Cat No. M0833, Santa Clara, CA, USA) at 4 °C overnight. The next day all samples were washed with IF solution three times, 20 min each, followed by addition of secondary antibody donkey anti goat IgG (Invitrogen; Cat No. A32849), anti-rabbit IgG (Invitrogen; Cat No. A31572), and anti-mouse IgG (Invitrogen; Cat No. A21202) for 2 h at room temperature in the dark. Finally, after three 5 min wash with DPBS, samples were exposed to 600mM DAPI in DPBS for 30 min and finally washed again DPBS three times, for 5 min each before imaging. Clearing was performed by soaking MultiCUBE samples overnight at room temperature with RapiClear^®^ 1.49 (SunJin Lab Co., Cat. No. RC149001; Hsinchu City, Taiwan) adding enough volume to cover the sample, then imaging was performed. All imaging was performed on Leica DMi8 widefield microscope (Leica Microsystems, Wetzlar, Germany) and processed on Imaris, version 10.1 (Bitplane, an Oxford Instruments Company, Zurich, Switzerland).

### Statistical analysis

All the statistical analysis was done in GraphPad Prism 8.3.0 software (GraphPad Software, Inc., Boston, MA, USA). Only continuous variables were analyzed. All data was presented using medians with 95%IC. Normality of the data was assessed to define the use of parametric or non-parametric statistical tests for further analysis. Overall repeated measures two-way ANOVA or mixed models with multiple comparison analysis correction was applied to compare multigroup analysis per plasma origin. An alfa value 0,05 was set for all conditions.

### Usage of generative AI and AI-assisted technologies in the writing process

During the preparation of this manuscript, the authors used OpenAI tools to enhance language clarity and readability during proof reading. All content was subsequently reviewed and edited by the authors, who take full responsibility for the final version of the publication.

## Supplementary Information

Below is the link to the electronic supplementary material.

Supplementary Material 1

Supplementary Material 2

Supplementary Material 3

## Data Availability

All data generated or analyzed during this study is available in accordance with reasonable requests to the corresponding author. Proteome dataset was provided as a supplementary material (Supplementary Table 2) which can be downloaded online: https://doi.org/10.1038/s41598-025-25523-1.
