# Supplementary material for: Hepatic vein-derived factors may affect pulmonary arteriovenous malformations after single ventricle palliation by modulating vascular cell behavior
Source: Sci Rep. 2025 Nov 24;15:41640. doi: 10.1038/s41598-025-25523-1 (PMC12644721; doi:10.1038/s41598-025-25523-1)
Supplement: Supplementary file 3 — Supplementary Material 3 [file 41598_2025_25523_MOESM3_ESM.pdf]

Supporting Information

Title

Hepatic Vein-Derived Factors May Affect Pulmonary Arteriovenous Malformations After Single Ventricle Palliation by Modulating Vascular Cell Behavior

Authors affiliation and contact information

Laura Yuriko González-Teshima[1,2], Keisuke Hakamada[1], Kozue Murata[1,2], Reiko Nakagawa[3], Shiro Baba[4], Yujiro Ide[1], Maiko Okamura[5], Akio Ikai[6], Tadashi Ikeda[1], Kenji Minatoya[1], Masaya Hagiwara[7], Masaya Ikegawa[5], Hidetoshi Masumoto[1,2*]

[1] Department of Cardiovascular Surgery, Graduate School of Medicine, Kyoto University, Kyoto, Japan
[2] Clinical Translational Research Program, RIKEN Center for Biosystems Dynamics Research, Kobe, Japan
[3] Laboratory for Cell-Free Protein Synthesis, RIKEN Center for Biosystems Dynamics Research, Kobe, Japan
[4] Department of Pediatrics, Graduate School of Medicine, Kyoto University, Kyoto, Japan
[5] Department of Life and Medical Systems, Doshisha University, Kyotanabe, Japan
[6] Division of Pulmonary Hemodynamics Research, Department of Clinical Research, Research Support Center, Shizuoka General Hospital, Shizuoka, Japan
[7] Human Biomimetic System RIKEN Hakubi Research Team, RIKEN Center for Biosystems Dynamics Research, Kobe, Japan

Corresponding author

Hidetoshi Masumoto, MD, PhD
Department of Cardiovascular Surgery, Graduate School of Medicine, Kyoto University
54 Kawara-cho, Shogoin, Sakyo-ku, Kyoto, 606-8507, Japan
Phone: +81-75-751-3784, Fax: +81-75-751-4960
E-mail: masumoto@kuhp.kyoto-u.ac.jp

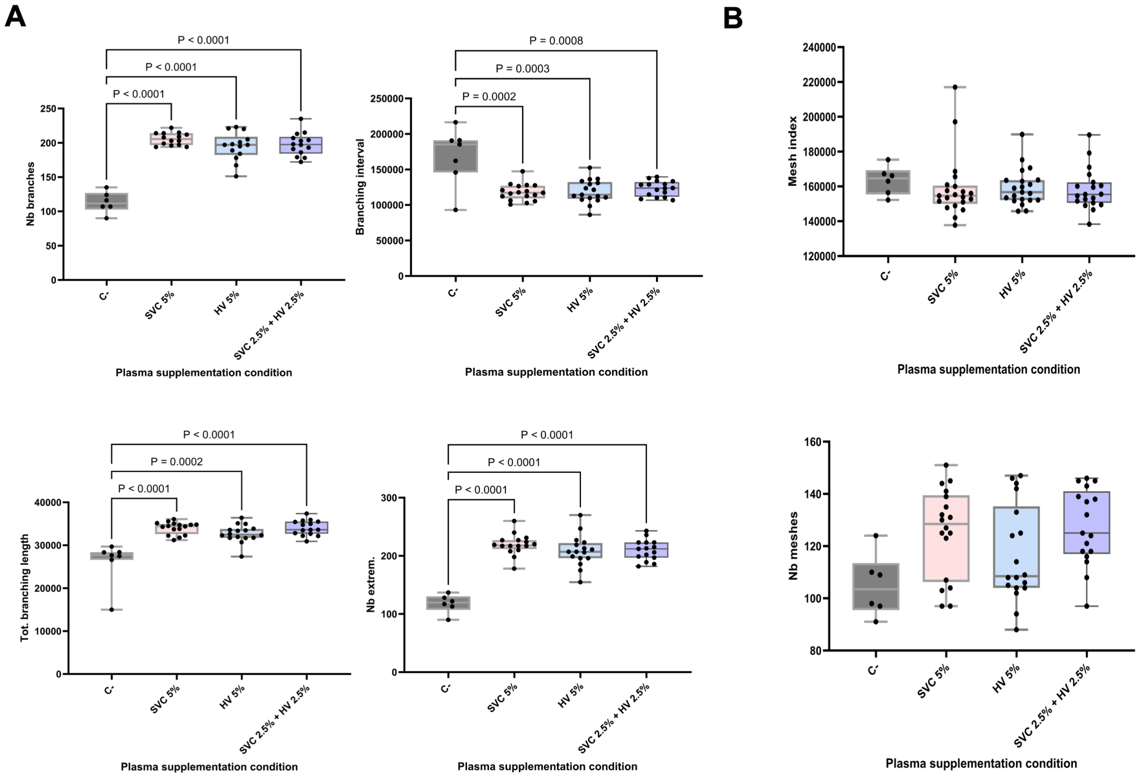

Supplementary figure 1. Arteriovenous tube formation assay analysis after 6 hours of culture A) Quantitative analysis of endothelial tubular network formation following 6 hours of culture under various plasma supplementation conditions. Nb. Branches, number of branches (top left); branching interval (top right); Tot. branching length, total branching length (bottom left); Nb. Extrem., number of extremities (bottom right). Superior vena cava (SVC), hepatic vein (HV). B) Mesh formation defined as the presence of enclosed loops in the network. Mesh index, number of meshes per unit area (top). Nb. Meshes, total number of meshes (bottom).

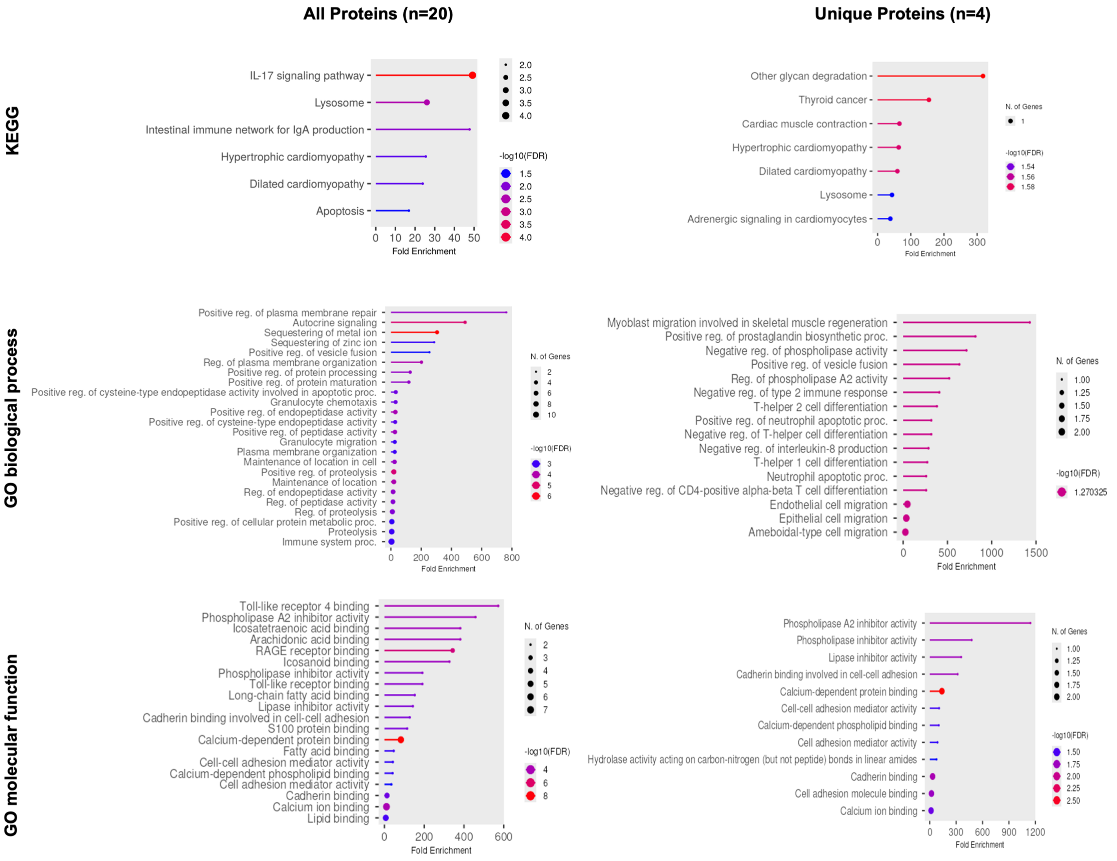

Supplementary figure 2. Functional enrichment analysis of proteins upregulated in superior vena cava (SVC) plasma ($p < 0.05$)

Functional gene ontology analysis including of all upregulated proteins in superior vena cava (SVC) (left column) and functional analysis including only uniquely expressed proteins in SVC (right column). Analysis are separated per database source: Kyoto Encyclopedia of Genes and Genomes (KEGG; top row), Gene Ontology resource biological process (GO biological process; middle row) and molecular function (GO molecular function; bottom row). Enrichment analyses were conducted using ShinyGO v0.82.

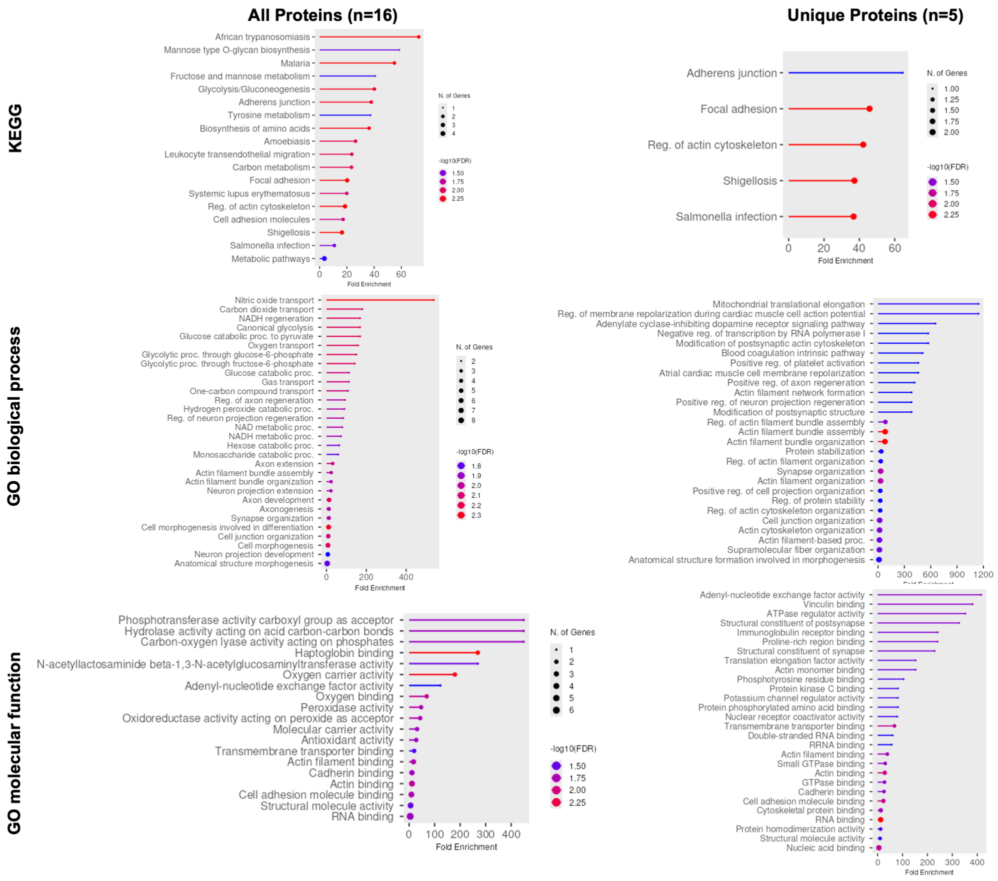

Supplementary figure 3. Functional enrichment analysis of proteins upregulated in hepatic vein (HV) plasma (p < 0.05)
Functional gene ontology analysis including of all upregulated proteins in HV (left column) and functional analysis including only uniquely expressed proteins in HV (right column). Analysis are separated per database source: Kyoto Encyclopedia of Genes and Genomes (KEGG; top row), Gene Ontology resource biological process (GO biological process; middle row) and molecular function (GO molecular function; bottom row). Enrichment analyses were conducted using ShinyGO v0.82.
